# Study of the Effect of Methyl Jasmonate Concentration on Aflatoxin B_1_ Biosynthesis by *Aspergillus parasiticus* in Yeast Extract Sucrose Medium

**DOI:** 10.1155/2009/842626

**Published:** 2009-12-06

**Authors:** Dido Maria Meimaroglou, Dia Galanopoulou, Panagiota Markaki

**Affiliations:** ^1^Department of Food Chemistry, School of Chemistry, University of Athens, Panepistimiopolis Zogra-fou, 15784 Athens, Greece; ^2^Laboratory of Biochemistry, School of Chemistry, University of Athens, Panepistimiopolis Zografou, 15784 Athens, Greece

## Abstract

Aflatoxin B_1_ (AFB_1_) is a carcinogenic metabolite produced by certain *Aspergillus* species on agricultural commodities. AFB_1_ biosynthesis is affected by jasmonic acid and also by its methylester (MeJA), a plant growth regulator derived from linoleic acid. This study reports the effect of MeJA on the growth of *A. parasiticus* and AFB_1_ output in yeast extract sucrose (YES) medium when added at three different concentrations; namely, 10^−2^ M, 10^−4^ M, and 10^−6^ M. AFB_1_ determination was performed by immunoaffinity and HPLC. MeJA at 10^−4^ and 10^−6^ M concentrations had no significant effect on mycelial growth but did affect AFB_1_ production after the 7th day of incubation; on the 12th day, AFB_1_ production was increased by 212.7% and 141.6% compared to the control samples (addition of 10^−6^ M and 10^−4^ M MeJA, resp.). Treatment of *A. parasiticus* cultures with 10^−2^ M MeJA inhibited mycelial growth and AFB_1_ production as well. These results suggest that the effect of MeJA on AFB_1_ biosynthesis by *A. parasiticus* depends on the MeJA concentration used.

## 1. Introduction

Aflatoxins are polyketide secondary fungal metabolites produced by the toxigenic strains of *Aspergillus flavus*, *Aspergillus parasiticus* and *Aspergillus nomius* [1–4], and they are known as potent carcinogenic, teratogenic as well as genotoxic mycotoxins. The most potent of the four naturally occurring aflatoxins is Aflatoxin B_1_ (AFB_1_) [5]. 

A variety of studies has been conducted in order to understand the process of crop contamination by aflatoxins. They all suggested that *Aspergilli *generally gain access to the plant seeds either through cracks generated by an environmental stress (heat or draft) or via insect damage [6, 7]. In addition, oily seeds are preferentially colonized in comparison to starchy ones [8]. Once the fungus has invaded the seed, it first destroys the lipid bodies, which are primarily composed of palmitic, oleic, and linolenic acids [9, 10]. Since there are many in vitro studies which have shown that lipid oxidation affects aflatoxin biosynthesis [11–14], it is of great interest to determine the mode of interaction between unsaturated fatty acids and their metabolites and aflatoxin production. Linoleic as well as linolenic acids can undergo a regio and stereospecific oxygenation [15] catalyzed by the widely distributed plant stress response enzyme lipoxygenase (LOX) [16] to yield 13S-hydroperoxy-cis-9-trans-11-octadecadienoic acid (13S-HPODE) and 13S-hydroperoxy-cis-9, trans-11, cis-15-octadecatrienoic acid (13S-HPOTE), respectively [17]. These fatty acid hydroperoxides are further converted through the octadecanoid pathway to jasmonates [18], a group of bioactive signaling compounds involved in different (multiple) aspects of plant response to their biotic and abiotic environment [19, 20].

In plants, jasmonates are synthesized as a response to systemic or localized signals like oligosaccharides released from fungi or plant cell walls during plant-pathogen interactions [21]. According to Pühler et al. [22], phytopathogenic bacterial species have developed specific methods to attack plant cells and to use plant molecules for their own growth; the bacterial genome research gave information on the distribution of bacterial secretion systems, which play a role in the interactions with plant cells. Weiler et al. [23] have reported that jasmonic acid levels increased rapidly in response to biotic and abiotic stress such as mechanical stress. Moreover, it has been reported that aflatoxins affect the amino acid uptake, enzymatic activities, germination as well as protein and nucleic acid synthesis in several plant systems. Recently, Ağar et al. [24] have reported that the levels of endogenous hormones (Gibberelic acid equivalents) decreased in *Zea mays* seeds treated with AFB_1_. In addition, natural elicitors were combined with methyl jasmonate (MeJA) to evaluate its effects on phytoalexin and AFB_1_ production in cotton plants [25]. 

In the case of *Aspergilli*, interactions between jasmonates, mycelial growth and aflatoxin production have been reported by several authors. These interactions are of great interest as they suggest that there is a mechanism involving plant LOX pathways that affect aflatoxin biosynthesis. It is interesting that both inhibition and stimulation of aflatoxin production by various LOX metabolites have been reported. Also, some hydroperoxy fatty acids may exert a stronger signalling influence on aflatoxin/sterigmatocystin (AF/ST) biosynthesis than on others. For example, aflatoxin biosynthesis by *A. parasiticus* was stimulated in synthetic medium containing a mixture of 30% 13S-HPODE and ~70% 13-HPODE although 13-HPODE has an inhibitory effect when tested alone. MeJA treatment at concentrations from 10^−6^ M to 10^−3^ M reduced AFB_1_ production by *A. flavus* grown on either Czapek yeast extract agar (CYA) medium or pistachios in storage [26]. On the contrary, Vergopoulou et al. [27] have reported that treatment with MeJA at a concentration of 10^−4^ M stimulated AFB_1_ production by *A. parasiticus *grown on yeast extract sucrose medium (YES). In a recent review, Holmes et al. [28] have underlined that lipoxygenase-generated signals, such as jasmonates, have both inhibitory and promoting effects on the AFB_1_ production by the aflatoxigenic *Aspergilli*.

The purpose of this study was to establish the significance and the consequences from the use of different MeJA concentrations on mold growth and AFB_1_ output under defined conditions.

## 2. Materials and Methods

### 2.1. Apparatus

A laminar flow (Telstar Bio IIA, Madrid, Spain), an autoclave (Selecta Autester-E Dry, PBI Milano, Italy), an incubator (WTB Binder, Tuttingen, Germany), and a centrifuge (Sorvall RC-5B, Norwalk, USA) were used during this study. HPLC was performed using a Hewlett-Packard 1050 (Waldborn, Germany) liquid chromatograph equipped with a JASCO FP-920 (Japan) fluorescence detector and an HP integrator 3395. The HPLC column used was a C18 Nova-Pak (60 $\overset{´}{Å}$, 4 *μ*m, 4.6 × 250 mm). The mobile phase for AFB_1_ determination [water+acetonitrile+methanol (20+4+3)] was filtered through Milipore HVLP filters (0.45 *μ*m) before use. Detection of the AFB_1_ hemiacetal derivative (AFB_2a_) was carried out at *λ*_ex_ = 365 nm and *λ*_em_ = 425 nm. The flow rate was 1 mL min ^−1^ and the retention time for AFB_2a_ was 8 minutes.

### 2.2. Reagents

The AFB_1_ standard was purchased from Sigma-Aldrich (St. Louis, MO, USA). The filters and the C18 Nova-Pak HPLC column were from Waters (Millipore, Milford, MA, USA). The Aflaprep immunoaffinity columns were from Rhone Diagnostics (Glasgow, UK). All other reagents and HPLC solvents were of HPLC grade (LABSCAN, Dublin, Ireland). Trifluoroacetic acid was purchased from Merck (Darmstadt, Germany). The purity of MeJA used was tested by GC analysis using a Hewlett-Packard gas chromatograph (equipped with a flame ionization detector) on a BPX70-coated fused-silica capillary column [29].

### 2.3. Media

*Aspergillus flavus *parasiticus agar (AFPA) was prepared by dissolving 4 g of yeast extract (Oxoid, Basingstoke, Hampshire, England), 2 g of bacteriological peptone (Oxoid), 0.1 g of ferric ammonium citrate, 0.2 mL of Dichloran (0.2% in ethanol, Fluka, Neu-Ulm, Switzerland), 0.02 g of chloramphenicol (Oxoid), and 3 g of agar (Oxoid) in 200 mL of distilled water, final pH 6.0–6.5 [30]. Czapek Dox agar (CZA) was prepared by dissolving 0.4 g of sodium nitrite, 0.1 g of potassium chloride, 0.1 g of magnesium sulfate, 0.002 g of ferric sulfate, 0.2 g of dipotassium phosphate, 6 g of sucrose, 3 g of agar, 0.002 g of zinc sulfate, and 0.001 g of copper sulfate in 200 mL distilled water, final pH 6.0–6.5. Yeast extract sucrose (YES) broth was prepared by dissolving 2 g of yeast extract and 15 g of sucrose in 100 mL distilled water, final pH 6.0–6.5 [30].

### 2.4. Preparation of Spore Inoculum

The aflatoxigenic strain *A. parasiticus* Speare (IMI 283883) utilized throughout this study was obtained from the International Mycological Institute (Engham, Surrey, UK). An inoculum was obtained by growing the mold on a slant of stock cultures of CZA, which were maintained at 5°C [31]. Spore inoculum was prepared by growing *A. parasiticus* on CZA for 7 days at 30°C, and spores were harvested aseptically using 10 mL of sterile 0.01 % (v/v) Tween 80 solution [32]. AFB_1_ carried over from the initial growth was minimized by centrifuging the spore suspension (1000 g for 1 min) and resuspending the biomass in 10 mL of sterile Tween 80 solution twice. Dilutions (10^−1^, 10^−2^, 10^−3^, 10^−4^) from the initial spore were prepared in sterile tubes containing 10 mL of 0.05% Tween 80 (v/v) suspension. The spore concentration was determined by the spread plate surface count technique, using 0.1 mL of each dilution on four AFPA plates [30, 33] after incubation at 30°C for 2 days. The population size was estimated by counting the single colonies from their reverse intense yellow/orange coloration. In order to obtain an inoculum containing 10^2^ conidia, plates with 10–100 colony forming units (cfu) were selected and the desired 10^2^ spore quantity used in this study was estimated. 

The quantity of 10^2^ spores flask^−1^ was chosen as it was the minimum concentration found in literature producing detectable amounts of AFB_1_ by *Aspergillus* [34].

### 2.5. Inoculation

Twelve flasks for each day of observation containing 10 mL of YES medium were inoculated with 10^2^ spores flask^−1^ of *A. parasiticus* in the appropriate volume from the selected dilution. MeJA in ethanol at final concentrations of 10^−2^ M, 10^−4^ M, and 10^−6^ M flask^−1^ was added into each of the three flasks for each day of observation. All flasks, control (simply ethanol) and treated with MeJA, were incubated under stationary conditions at 30°C. Immediately after autoclaving for 30 minutes at 115°C as it is suggested for safety reasons [35], the mycelial growth was determined and AFB_1_ was assayed on days 0, 3, 7, 9, 12, and 15 of incubation. The experiment was repeated in triplicate.

### 2.6. AFB_1_ Determination

The content of each flask (containing the fungus in YES medium) was mixed with 30 mL of methanol and wellshaken for 10 min. After filtration, an aliquot of 1 mL from each flask was used for AFB_1_ analysis. The 1 mL aliquot from the filtrate was mixed with 10 mL distilled water. The mixture was transferred onto an Aflaprep immunoaffinity column and washed twice with 10 mL of distilled water (flow rate: 6 mL min^−1^). The column was then allowed once more to dry by passing air through it. AFB_1_ was eluted with 2 mL of acetonitrile (flow rate: 0.3 mL min^−1^). Before derivatization, the eluate was evaporated to dryness on a water bath under a gentle steam of nitrogen [36].

### 2.7. Derivatization and HPLC Analysis

A derivative of AFB_1_ (AFB_2a_, hemiacetal of AFB_1_) was prepared by adding 200 *μ*L of hexane and 200 *μ*L of trifluoroacetic acid to the evaporated solution of AFB_1_ eluate, heating for 10 min at 40°C in a water bath, evaporating to dryness under nitrogen, redissolving in an appropriate volume of water-acetonitrile (9 : 1) to give an AFB_1_ concentration of <10 ng mL^−1^ and analyzing by HPLC (volume injected: 20 *μ*L). AFB_2a_ shows enhanced fluorescence compared to AFB_1_ [36].

### 2.8. Determination of Mycelial Mass

After extraction, mycelia were filtered through filters that were previously dried (24 h at 80°C) and weighed. The mycelium was washed with distilled water and allowed to dry for 24 h at 80°C. The dry weight of the mycelium was then determined [37].

### 2.9. Statistical Analysis

Data were analyzed by one-way and two-way analysis of variance. The mean differences which are significantly different were examined by using the Tukey test [38].

## 3. Results and Discussion

The analytical protocol for AFB_1 _determination in YES medium was previously in-house characterized in detail by Leontopoulos et al. [39]. The recovery of the method was found to be 90.9% and the detection limit, based on a signal-to-noise ratio of 3 : 1 at the retention time (8 min) of the derivatized AFB_1_ (AFB_2a_), was 0.2 ng flask^−1^ corresponding to 0.02 ng mL^−1^ YES medium.

A satisfactory linear relationship was established between different quantities (1, 2.5, 5 *μ*g) of AFB_1_ spiked in 10 mL of YES medium and quantities recovered (*y* = 0.909*x* + 0.3, *r* = 0.999).

### 3.1. The Effect of MeJa on *A. parasiticus* Growth

YES medium is an optimum medium for *A. parasiticus* growth and AFB_1_ biosynthesis [40]. In the present study, *A. parasiticus* was used because AFB_1_ production is a more stable trait in this fungus than in *A. flavus* [41]. In addition, AFB_1_ was studied throughout this study as it is the most potent mycotoxin and it is usually produced at the highest levels by toxigenic strains [42]. 

We studied the effect of MeJA at final concentrations 10^−6^ M, 10^−4^ M, and 10^−2^ M on both mycelial growth of *A. parasiticus* in YES medium and AFB_1_ production. It must be mentioned that 10^−4^ M is the concentration of MeJA which has been found to be effective as a postharvest treatment for suppressing the decay caused by *Botrytis cinerea* on strawberries, for reducing the decay by *Penicillium digitarum* in grapefruit as well as for reducing microbial contamination in celery and peppers [43]. Gogala [44] has also reported that jasmonate was highly active at medium concentrations (10^−6^ M to 10^−4^ M), but inhibition of the mycorrhizal growth has been observed at lower concentrations (~10^−7^ M). To our knowledge, the 10^−2^ M concentration of MeJA has not yet been tested.

The mycelial growth of nontreated and MeJA-treated *A. parasiticus* in YES medium is shown in Table 1. The maximum growth of the mold was observed on the 7th day after inoculation for the control samples (352.5 mg flask^−1^) as well as for the samples treated with MeJA at concentrations 10^−6^ M (374.8 mg flask^−1^) and 10^−4^ M (359.5 mg flask^−1^). On the contrary, no visible mycelial growth by *A. parasiticus* or measurable amount of the fungus was observed in samples treated with MeJA 10^−2^ M during the whole period of incubation (15 days).

The statistical analysis by using one-way Anova applied to all groups, with or without MeJA treatment, showed that the *F*_exp_ = 7.40 was higher than the *F*_theor_ = 3.05 for df 3, 20. Therefore, the variation of mycelial growth between the four groups is statistically significant (*P* < .05). It must be added, however, that the statistically significant variation between the four groups of samples may be due to just one of the four groups, probably to the samples treated with 10^−2^ M MeJA. The comparison of variances of the other three groups (control, MeJA 10^−6^ M, MeJA 10^−4^ M) showed that, at 0.05 level, the differences between the mycelial growth for the three groups are not significant and that the 10^−6^ M and 10^−4^ M MeJA concentrations had no apparent effect on *A. parasiticus* growth. 

 These results are in agreement with Vergopoulou et al. [27] who only studied the 10^−4^ M MeJA concentration and showed that no effect on the mycelial growth of *A. parasiticus* was observed. Goodrich-Tanrikulu et al. [26] also reported that MeJA concentrations ranging from 10^−3^ M to 10^−8^ M had no apparent effect on the mycelial growth of *A. flavus*.

### 3.2. MeJa Effect on AFB_1_ Production in YES Medium

In this study, AFB_1_ production was measurable from day 0 of the incubation (0.002 *μ*g AFB_1_ flask^−1^, Table 2)

This AFB_1_ occurrence was due to the inoculation of samples with 10^−2^ conidia of *A. parasiticus *per flask. In the case of cultures treated with 10^−2^ M MeJA, the AFB_1_ amounts are negligible in comparison to AFB_1_ production in samples treated with MeJA at concentrations 10^−6^ M and 10^−4^ M as well as in control samples during the whole period of incubation. These traces of AFB_1_ are probably due to the conidia of *A. parasiticus*, which survived in spite of the inhibition of the fungus growth by the MeJA at this concentration. Treatment with MeJA at the highest concentration (10^−2^ M) reduced AFB_1_ production against control by 99.6% to 99.9% during the incubation while, on the 15th day of observation, AFB_1_ was not detectable ( < *D L* = 0.02 ng mL^−1^ YES). This was due to the inhibition of *A. parasiticus* growth by MeJA. 

**Figure 1:**
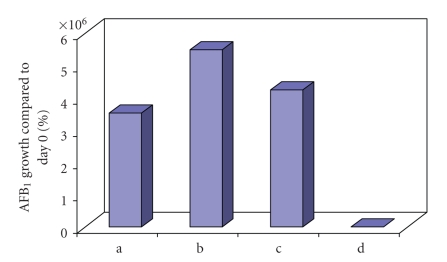
% maximum AFB_1_ production by *A. parasiticus* in YES medium compared to day 0 in: (a) control samples on day 15, (b) samples treated with MeJA (10^−6^ M) on day 12, (c) samples treated with MeJA (10^−4^ M) on day 12, and (d) samples treated with MeJA (10^−2^ M) on day 9.

In the case of samples treated with 10^−6^ M and 10^−4^ M MeJA, treatment enhanced AFB_1 _ biosynthesis by *A. parasiticus*, while maximum AFB_1_ production was observed on the 12th day. This production was 5.5 × 10^7^ and 4.3 × 10^7^ times higher compared to samples on day 0, respectively, as shown in Figure 1. AFB_1_ production was also observed in control samples but, in this case, maximum production (3.5 × 10^7^) was revealed on the 15th day (Figure 1). Under the same conditions, treatment with 10^−2^ M MeJA resulted in only 3 × 10^3^ (9th day) AFB_1_ maximum production compared to day 0.

As shown in Table 2, in the samples treated with MeJA at concentration 10^−6^ M, AFB_1_ output was stimulated after the 9th day of incubation while maximum production was observed on the 12th day (109.91 *μ*g flask^−1^), and thus reaching 212.8% of the control. In the samples treated with MeJA at concentration 10^−4^ M, AFB_1_ production was also stimulated after the 9th day and reached on the 12th day 84.91 *μ*g AFB_1_ flask^−1^, which corresponds to 141.6% of the control. Concerning 10^−4^ MeJA concentration, the results are in agreement with Vergopoulou et al. [27] who previously reported that this MeJA concetration stimulated AFB_1_ production by *A. parasiticus* after the 7th day of incubation, although Goodrich-Tanrikulu et al. [26] showed that aflatoxin production by *A. flavus *was inhibited at all MeJA concentrations tested, which ranged from 10^−3^ M to 10^−8^ M. It must be added that according to De Luca et al. [14] and Fabbri et al. [11], aflatoxin production increased 50 to 200 times after treatment of 10-day-old cultures of *A. parasiticus* and *A. flavus* with a mixture of linoleic acid and soybean LOX1. Furthermore, according to Greene-McDowelle et al. [45], although some LOX products have antifungal activity, other LOX products influence aflatoxin production while they have little influence on the fungal growth. It is obvious that the results of this study support a similar mechanism for the MeJA action. 

Our results were confirmed by the two-way Anova statistical analysis.The Null Hypothesis is that there are no significant differences between the aflatoxin output at different MeJA concentrations. The value of *F*_exper_ = 0.238.46 greatly exceeds that one tabulated at *P* = .05, namely, about 3.8 at df 3, 40. The Null Hypothesis is therefore rejected and it is concluded that different MeJA concentrations do affect the aflatoxin output. The second Null Hypothesis is that there are no significant differences between the aflatoxin outputs on different days of incubation. The value of *F* = 32.34 exceeds the tabulated value at *P* = .05 of 4.21 for df 9, 40. The Null Hypothesis is therefore rejected and it is concluded that incubation days do affect the aflatoxin output. The third Null Hypothesis is that there is no interaction between different MeJA concentrations and days of incubation, which influences the aflatoxin outputs. The calculated value of *F* = 11.68 exceeds the tabulated one at *P* = .05, namely, 2.039 at df 12, 40. We therefore reject the Null Hypothesis once more and conclude that there is an interaction between different MeJA concentrations and days of incubation, which influences aflatoxin output. These results revealed the possibility of the existence of different mechanisms, by which MeJA influences AFB_1_ biosynthesis when different concentrations are used.

Burow et al. [42] have already reported that treatment with 9S-HPODE increased or decreased aflatoxin production depending on the concentration tested. In addition, 9S-HPODE induced prolonged accumulation of transcripts of the aflatoxin/sterigmatocystin biosynthetic genes. Several jasmonates have also been shown to activate genes encoding antifungal proteins such as thionin [46], osmotin [47], as well as genes involved in phytoalexin biosynthesis [48]. In addition, cultures of *A. parasiticus* and *A. flavus* produce amounts of aflatoxins, which decrease during continued incubation of the cultures [49]. In this case, the authors reported that molds, which are capable of producing aflatoxin, may also degrade them. Doyle and Marth [50] observed that the ability of *Aspergilli* to degrade aflatoxins was dependent on the time of incubation; mycelia aging 8 to 10 days old were most effective in degrading AFB_1_. 

According to Sweeney and Dobson [4], AFB_1_ biosynthesis is regulated at the level of transcription of genes involved in the aflatoxin biosynthetic pathway. These genes include two fatty acid synthase genes, a polyacetide synthase gene as well as the ord-1-gene, which encodes a cytochrome P-450 type monooxygenase, putatively responsible for the conversion of O-methylsterigmatocystin to aflatoxin. This monooxygenase is also involved in the degradative activity of *A. flavus* [51]. Thus, when jasmonates are exogenously applied to plant tissues, they exert either inhibitory or promoting effects in growth and developmental processes [19] and this finding concerns both the *Aspergilli* growth on plants as well as AFB_1_ biosynthesis.

The effectiveness of MeJA suggests a potential use in the postharvesting control of aflatoxin production in susceptible commodities like pistachios [26]. Moline et al. [52] also reported that MeJA can be applied effectively as a postharvest treatment to supress grey mold rot caused by *Bacillus cinerea* in strawberry. Markaki et al. [53], however, showed that when olives were treated with MeJA at different concentrations, AFB_1_ production was concentration-dependent (AFB_1_ either decreased or increased) in both olives inoculated with *A. parasiticus* and in noninoculated samples. It should be mentioned, in this case, that olives are not a suitable substrate for AFB_1_ biosynthesis [39].

 In conclusion, in this study, it is shown that the plant growth regulator MeJA at a concentration of 10^−2^ M inhibits *A. parasiticus* growth on YES medium, and consequently, AFB_1_ production is insignificant. As far as lower concentrations are concerned, although in this study stimulation is reported, there are conflict results in literature concerning the effect of MeJA on AFB_1_ production. Therefore, it appears to be very important to identify the conditions under which the use of MeJA could be effective in preventing the biosynthesis of AFB_1 _ mainly in products destined for long storage.

## Figures and Tables

**Table 1:** Mycelial growth (mg flask^−1^) of *A. parasiticus* in YES medium.

MeJA addition				
	0^(a)^	10^−6 ^M^(b)^	10^−4 ^M^(c)^	10^−2 ^M^(d)^
Dry weight of mycelium				
Days	mg flask^−1^	mg flask^−1^	mg flask^−1^	mg flask^−1^
	(±SD)	(±SD)	(±SD)	(±SD)
0	0	0	0	0
3	282.7 (±17.7)	290.8 (±5.0)	259.2 (±5.0)	NM^e^
7	352.5 (±9.9)	374.8 (±5.1)	359.5 (±5.8)	NM
9	314.9 (±13.5)	341.8 (±9.5)	321.6 (±14.4)	NM
12	272.4 (±4.7)	296.3 (±1.8)	285.4 (±17.4)	NM
15	224.6 (±23.54)	249.8 (±5.4)	233.3 (±21.5)	NM

^(a)^Without MeJA addition (control); ^(b)^addition of 0.0022 mg MeJA flask^−1^; ^(c)^addition of 0.227 mg MeJA flask^−1^; ^(d)^addition of 22.67 mg; no visible mycelial growth was observed during the whole period of observation; ^(e)^non measurable.

**Table 2:** AFB_1_ production (*μ*g flask^−1^) by *A. parasiticus* in YES medium.

MeJA addition				
	0^(a)^	10^−6^ M^(b)^	10^−4^ M^(c)^	10^−2^ M^(d)^
AFB_1_ production				
Days	*μ*g flask^−1^(± SD)	*μ*g flask^−1^(± SD)	*μ*g flask^−1^(± SD)	*μ*g flask^−1^(± SD)
0	0.002 (±0)	0.002 (±0)	0.002 (±0)	0.002 (±0)
3	40.17 (±4.31)	44.94 (±5.39)	23.45 (±5.76)	0.019 (±0.002)
7	56.18 (±15.86)	72.44 (±4.17)	69.01 (±17.06)	0.051 (±0.005)
9	29.80 (± 8.11)	60.16 (±9.26)	48.00 (±6.30)	0.063 (±0.006)
12	35.14 (±9.37)	109.91 (±10.78)	84.91 (±7.66)	0.061 (±0.004)
15	70.57 (±4.69)	89.58 (±6.82)	58.51 (±11.45)	ND^(e)^

^(a)^Without MeJA addition (control); ^(b)^addition of 0.0022 mg flask^−1^; ^(c)^addition of 0.227 mg flask^−1^; ^(d)^addition of 22.67 mg flask^−1^; ^(e)^ND: not detected.
